# Geometric mechanics of kiri-origami-based bifurcated mechanical metamaterials

**DOI:** 10.1098/rsta.2024.0010

**Published:** 2024-10-07

**Authors:** Yanbin Li, Caizhi Zhou, Jie Yin

**Affiliations:** ^1^ Department of Mechanical and Aerospace Engineering, North Carolina State University, Raleigh, NC 27606, USA

**Keywords:** origami and kirigami, reconfigurable hierarchical mechanical metamaterials, kinematic bifurcation, tunable Poisson’s ratios, stiffness anisotropy

## Abstract

We explore a new design strategy of leveraging kinematic bifurcation in creating origami/kirigami-based three-dimensional (3D) hierarchical, reconfigurable, mechanical metamaterials with tunable mechanical responses. We start from constructing three basic, thick, panel-based structural units composed of 4, 6 and 8 rigidly rotatable cubes in close-looped connections. They are modelled, respectively, as 4R, 6R and 8R (R stands for revolute joint) spatial looped kinematic mechanisms, and are used to create a library of reconfigurable hierarchical building blocks that exhibit kinematic bifurcations. We analytically investigate their reconfiguration kinematics and predict the occurrence and locations of kinematic bifurcations through a trial-correction modelling method. These building blocks are tessellated in 3D to create various 3D bifurcated hierarchical mechanical metamaterials that preserve the kinematic bifurcations in their building blocks to reconfigure into different 3D architectures. By combining the kinematics and considering the elastic torsional energy stored in the folds, we develop the geometric mechanics to predict their tunable anisotropic Poisson’s ratios and stiffnesses. We find that kinematic bifurcation can significantly effect mechanical responses, including changing the sign of Poisson’s ratios from negative to positive beyond bifurcation, tuning the anisotropy, and overcoming the polarity of structural stiffness and enhancing the number of deformation paths with more reconfigured shapes.

This article is part of the theme issue ‘Origami/Kirigami-inspired structures: from fundamentals to applications’.

## 1. Introduction

Recently, mechanical metamaterials have attracted increasing research interest owing to their unprecedented physical properties [1–7]. These unusual properties enable new functionalities for shape morphing [8–10], robotic applications [11–13] and mechanical computing [14–18]. The design space and fabrication of mechanical metamaterials have been significantly extended with recent advances in artificial intelligence [17,19–22] and advanced additive manufacturing techniques [23–25]. Among the extensive design strategies, the origami and kirigami approach [26,27] opens a new avenue in creating reconfigurable mechanical metamaterials by utilizing the unique folding or cutting patterns for shape morphing and deployment after fabrication. The approach unlocks unique mechanical behaviours via structural reconfiguration or deployment, such as high stretchability [28–33], tunable negative Poisson’s ratio [34–37], instabilities and multistabilities [38–42], programmable shape morphing [26,43–46], topologically tunable mechanical responses [47] and encoded machine-like intelligence [48–52].

Recent works on kirigami and origami metamaterials leverage periodic and/or irregular cut and fold patterns [26,29,32,43,45,46] to create two-dimensional (2D) networks [30,32,35] or three-dimensional (3D) structural forms. The prescribed folding or cutting patterns determine the deformation and shape changes governed by mechanical properties. However, once fabricated, most of the origami and kirigami metamaterials can only transform into one (or at best a few) deterministic structural configurations owing to a low number of degrees of freedom (d.f.) [26,30,31,43,45,46,53,54]. Consequently, the tunability of their mechanical behaviours is rather limited without changing the designed cut or fold patterns. To address this limitation, recently, new forms of cube-based 3D reconfigurable origami and kirigami metamaterials have been proposed by means of connecting deformable or rigid cubes with elastic and rotatable line hinges at the cube edges [55–57]. The structural d.f. are determined by how a certain number of cubes are connected in open or closed loops and then tessellated in planar or in 3D space, i.e. the 2D or 3D folding or cutting patterns. This largely releases the constrained d.f. in conventional 2D and 3D origami/kirigami metamaterials, allowing multiple d.f. to transform into multiple and even hundreds of configurations without changing the prescribed fold or cut patterns after fabrication [55–57]. In particular, the enhanced d.f. in origami/kirigami metamaterials can be enabled by kinematic bifurcations that can branch into multiple reconfiguration paths, leading to enhanced reconfigurability [56]. However, how such kinematic bifurcations affect the mechanical behaviours of 3D origami/kirigami metamaterials remains largely unexplored. In this work, inspired by our recently proposed concept of hierarchical origami metastructures [58], we report leveraging kinematic bifurcations for tunable anisotropic structural stiffness and Poisson’s ratios in a new library of kirigami- and origami-based hierarchical mechanical metamaterials. We show that the kinematic bifurcations are attributed to both the structural hierarchies and the looped internal connections of their folds. Comparing with existing works [59–61], our work advances in terms of (i) coupling kinematic bifurcations in both the origami–kirigami inspired structural units and their 3D tessellated hierarchical metamaterials for enhanced reconfigurability, (ii) demonstrating the high efficiency of structural hierarchy on increasing the reconfiguration d.f. of the building blocks, (iii) overcoming the polarity and singularity on tuning anisotropic structural stiffness and Poisson’s ratio via kinematic bifurcation and (iv) significantly enhancing the deformation paths with enormous number of reconfigured shapes in periodic 3D metamaterials via kinematic bifurcation.

The rest of this article is organized as follows. The concept of the bifurcated hierarchical mechanical metamaterials is introduced in §2. Then, we systematically investigate the construction and reconfiguration features (kinematics) of the hierarchical building blocks. We summarize the theoretical and numerical modelling results in §3. Furthermore, we study the geometric mechanics of our proposed bifurcated mechanical metamaterials. The analytical results of the tunable structural stiffness and Poisson’s ratios are present in §4. Finally, further remarks are made in §5.

## 2. Construction of cube-based structural units

**Figure 1.**
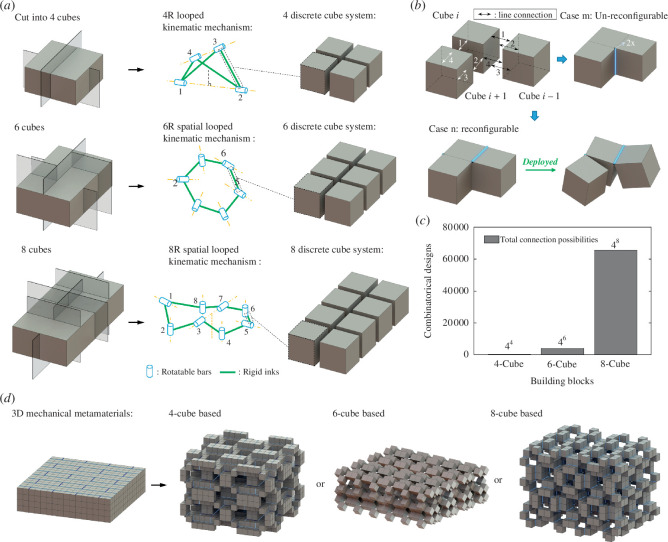
(*a*) Schematics of constructing three categories of cube-based structural units via plane cutting. (*b*) Schematic illustration of the combinatorial designs by selectively connecting the facing edges of the adjacent cubes. (*c*) The number of combinatorial designs for (*b*) and (*c*), and three of their composed hierarchical mechanical metamaterials.


Figure 1 illustrates the construction process of our proposed hierarchical mechanical metamaterials. Following our previous work on the modular kirigami approach [55,56], we start from constructing the structural units by dissecting three thick bulk solids (figure 1*a*, left) using non-through plane cuts into spatial kinematic cube-based systems (figure 1*a*, middle). The number of internal line hinges or folds (uncut margins) is equal to the quantity of the cube-shaped structural elements, i.e.

(2.1) $$N_{fold}=N_{element}.$$

Thus, each pair of any two neighbouring cubes is connected with only one line fold at the facing edges, i.e.


(2.2) $$n_{fold}=1,$$

where $n_{fold}$ represents the number of line folds between two adjacent structural elements. After dissection, there remain three categories of structural units made of 4 cubes, 6 cubes and 8 cubes.

The merits of partitioning the solids by following the rules constrained by equations (2.1) and (2.2) are reasoned by

1. Introducing the fewest number of structural constraints in the structural units;
2. Enabling the structural units with the potential maximum quantity of reconfiguration paths;
3. Forming the structural units into over-constrained looped spatial kinematic mechanisms that could potentially induce kinematic bifurcations discussed later.

For the 4-, 6- and 8-cube structural units, the cubes are assumed to be rigid, and line folds can only rotate. Thus, they can be considered as 4R, 6R and 8R (R stands for revolute joint) spatial looped kinematic mechanisms, respectively. The rigid cubes act as rigid links and the line folds act as rotational bars with their reconfiguration kinematics predicted by the Denavit–Hartenberg (D–H) rule [53,54,58,62].

The multiple edges of the cubes enable the combinatorial connections with their neighbouring facing cubes (figure 1*b*). For example, for the three adjacent cubes shown in figure 1*b*, each two facing cubes accommodate four pairs of edges for one edge connection in between as one line fold in equation (2.2). Thus, for a structural unit with *l* cubes, it allows a total number of combinatorial connections, i.e. designs (figure 1*c*),

 (2.3) $$N_{Fold\_connection}=4^{l}\;(l=4,6\;\;and\;\;8).$$

Essentially, these fold connections determine their folding patterns and their kinematic transformation. Figure 1*b* shows that despite the large number of combinatorial designs, not all the fold connections can undergo kinematic structural reconfiguration.

Next, we explore, using these structural units, the construction of various bifurcated building blocks in a hierarchical way [58] and then describe how to create new 3D reconfigurable hierarchical mechanical metamaterials with preserved kinematic bifurcations and multiple transformation paths (figure 1*d*).

## 3. General rule on modelling hierarchical reconfigurable building blocks

In this section, we first study the combinatorial designs and their reconfiguration kinematics through a trial and correction numerical method. Then, by introducing structural hierarchy in the building blocks, we explore the general design rules of constructing hierarchical building blocks and the optimal designs for creating hierarchical mechanical metamaterials.

### (a) General model on the reconfiguration kinematics of structural units

Given the looped kinematic mechanism structural features, the reconfiguration kinematics of the cube-based structural units can be accurately described by the rotation angle, *θ,* of the folds using the D–H rule [62], i.e.


(3.1) $$\prod_{q=1}^{l}T_{q}=I,$$

where **T** is a homogeneous matrix with an explicit form as


(3.2) $$T=T\left(\alpha ,\theta ,\;L,s\right)=\left[\begin{array}{cccc} 1 & 0 & 0 & 0\\ -L & cos\theta & sin\theta & 0\\ -Lsin\alpha & -cos\alpha sin\theta & cos\alpha cos\theta & sin\alpha \\ -scos\alpha & sin\alpha sin\theta & -sin\alpha cos\theta & cos\alpha \end{array}\right],$$

where *L*, *α*, *θ* and *s* are the distance between two adjacent rotatory folds, the angle between adjacent rotatory folds, the angle between rigid links and the distance between rigid links, respectively; **I** is a 4 × 4 homogeneous identical matrix with


(3.3) $$I=\left[\begin{array}{cccc} 1 & 0 & 0 & 0\\ 0 & 1 & 0 & 0\\ 0 & 0 & 1 & 0\\ 0 & 0 & 0 & 1 \end{array}\right].$$

Generally, kinematic bifurcation (a reconfiguration singular point from where new reconfiguration paths occur in addition to the original one) can occur in the looped kinematic mechanism. Thus, based on equations (3.1) and (3.2), a trial-correction numerical method can be used to predict the reconfiguration kinematics of the structural units by considering the potential bifurcation. Consequently, the quantity of the reconfiguration d.f., $N_{DoFs}$, can be obtained in terms of [53,56,57]:


(3.4) $$N_{DoFs}=N_{Reconf\_path},$$

where $N_{Reconf\_path}$ is the number of the reconfiguration paths.

During analysis, an initial value of *θ*_0_ in the fold rotation angle should be assigned for the studied looped kinematic mechanisms (note that given the structural reconfiguration features, at least two hinge rotation angles are non-zero). Since the assigned initial rotating values are generally not the genuine solutions, substituting *θ*_0_ into the D–H rule (equation (3.1)) will generate errors **E**, i.e.


(3.5) $$\prod_{q=1}^{l}T_{q}\left(\theta_{0}\right)=I-E.$$

To obtain the genuine solutions, the linear perturbation method can be applied to eliminate the errors.

Specifically, *θ*_0_ can be re-decomposed into two parts, the error-free part *θ*_*c*0_ (genuine solution) and the error part *δθ*_*E*0_, i.e.


(3.6) $$\theta_{0}=\theta_{c0}+\delta \theta_{E0}.$$

Substituting equation (3.6) into equation (3.5) gives


(3.7) $$\prod_{q=1}^{l}T_{q}\left(\theta_{c0}+\delta \theta_{E0}\right)=I-E.$$

Then conducting a Taylor expansion and neglecting the higher-order terms (*δθ*_*E*0_)^*n*^ (*n* is an integer with *n* > 1), we have


(3.8) $$T_{q}\left(\theta_{c0}+\delta \theta_{E0}\right)=T_{q}\left(\theta_{c0}\right)+T_{q}^{\prime }\left(\theta_{c0}\right)\delta \theta_{E0},$$

where $T_{q}^{\prime }\left(\theta_{c0}\right)=\partial T_{q}/\partial \theta_{c0}.$

Combining equation (3.8) and equation (3.7) gives


(3.9) $$\prod_{q=1}^{l}T_{q}\left(\theta_{c0}\right)+T_{1}^{\prime }\left(\theta_{c0}\right)\prod_{q=2}^{l}T_{q}\left(\theta_{c0}\right)\delta \theta_{E0}+\cdots T_{l}^{\prime }\left(\theta_{c0}\right)\prod_{q=1}^{l}T_{q}\left(\theta_{c0}\right)\delta \theta_{E0}=I-E.$$

Moreover, given that the rotation of the folds are internal, we can decompose the transformation matrix into a constant translational part $T_{q}^{T}$ and a varying rotational part $T_{q}^{R}$. Accordingly, we have


(3.10) $$T_{q}=T_{q}^{T}\cdot T_{q}^{R},$$

where the rotational matrix $T_{q}^{R}$takes an explicit form as


(3.11) $$T_{q}^{R}=T\left(\theta \right)=\left[\begin{array}{cccc} 1 & 0 & 0 & 0\\ 0 & 1 & 0 & 0\\ 0 & 0 & cos\theta & -sin\theta \\ 0 & 0 & -sin\theta & cos\theta \end{array}\right].$$

Thus, based on equation (3.10), we can rewrite equation (3.11) as


(3.12) $$\prod_{q=1}^{l}T_{q}\left(\theta_{c0}\right)+T_{1}^{T}\left(\theta_{c0}\right){\left[T_{1}^{R}\left(\theta_{c0}\right)\right]}^{\prime \prod_{q=2}^{l}T_{q}\left(\theta_{c0}\right)}\delta \theta_{E0}+\;\cdots T_{l}^{T}\left(\theta_{c0}\right)\left[T_{l}^{R}\left(\theta_{c0}\right)\right]\prime \prod_{q=1}^{l}T_{q}^{T}\left(\theta_{c0}\right)T_{q}^{R}\left(\theta_{c0}\right)\delta \theta_{E0}=I-E.$$

Since the rotation angle $\theta_{c0}$ is the error-free solution satisfying equation (3.3), we can simplify equation (3.12) as


(3.13) $$T_{1}^{T}\left(\theta_{c0}\right){\left[T_{1}^{R}\left(\theta_{c0}\right)\right]}^{\prime \prod_{q=2}^{l}T_{q}\left(\theta_{c0}\right)}\delta \theta_{E0}+\;\cdots T_{l}^{T}\left(\theta_{c0}\right)\left[T_{l}^{R}\left(\theta_{c0}\right)\right]\prime \prod_{q=1}^{l}T_{q}^{T}\left(\theta_{c0}\right)T_{q}^{R}\left(\theta_{c0}\right)\delta \theta_{E0}=-E.$$

Introducing a coefficient matrix **C**, the above equation can be rewritten as


(3.14) $$\prod_{q=1}^{l}C_{q}\delta \theta_{E0}=-E,$$

$$with\;\;\;C_{q}=T_{1}^{T}\left(\theta_{c0}\right)T_{1}^{R}\left(\theta_{c0}\right)\cdot \cdots \cdot T_{q}^{T}\left(\theta_{c0}\right)\left[T_{q}^{R}\left(\theta_{c0}\right)\right]\prime \cdot \cdots \cdot T_{l}^{T}\left(\theta_{c0}\right)T_{l}^{R}\left(\theta_{c0}\right).$$

Finally, the least-square solution can be used for equation (3.15) to determine the minimal correcting angles *δθ_Ε_*_0_. Then, the error-free prediction rotating angles *θ*_*c*0_ can be obtained using


(3.15) $$\theta_{c0}=\;\theta_{0}-\delta \theta_{E0}.$$

It should be noted that different sets of initial values should be tried by combinatorically selecting 2 to *q* rotation angles since kinematic bifurcation represents more than one reconfiguration path. In the following, we analyse the reconfiguration features of our proposed three different types of structural units.

### (b) 4-cube-based structural unit and the hierarchical building block #1

**Figure 2.**
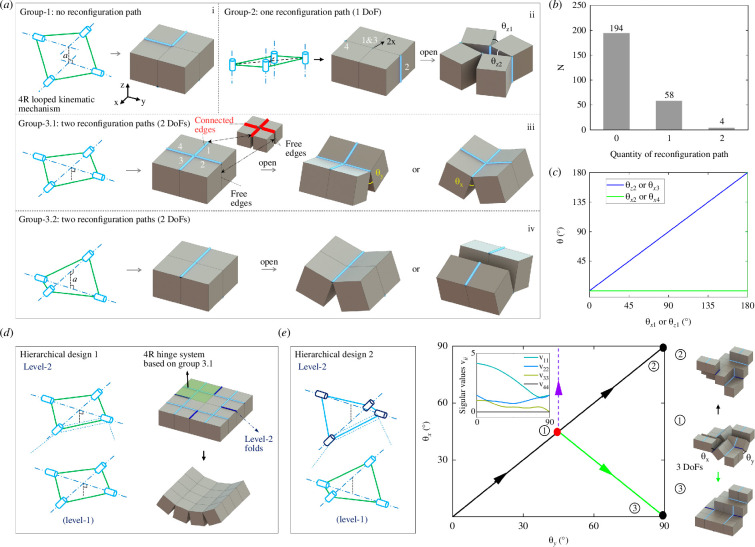
Reconfiguration details of 4-cube structural units and the first hierarchical building block. (*a*) Three groups of 4-cube units with 0, 1 and 2 reconfiguration paths in (i), (ii) and (iii), respectively. Red lines are the connected edges. (*b*) The number of combinatorial designs versus the quantity of the reconfiguration paths. (*c*) The linear angle relations of folds in the simple structural topology. (*d–e*) Two representative examples of hierarchical building blocks based on the 4-cube structural units. The kinematic bifurcation of angle changes and variations of singular values *v*_*ii*_ (insets) during its reconfiguration from ① to ③ are shown to the right of (*e*).


By calculating the number of reconfiguration paths, we can classify the 4-cube-based units into three groups. The first group is totally constrained without any reconfiguration path. We attribute this to its least structural symmetries. We present one representative fold pattern with its equivalent looped kinematic mechanism in figure 2*a*(i). Owing to the structural asymmetry, there are always constraints that do not allow any form of fold rotations, i.e.

(3.16) $$\theta_{xk}=\;\theta_{yk}=\theta_{zk}\equiv 0,\;\left(k=1\;or\;2\right).$$

The second group exhibits only one reconfiguration path. Two different types of fold patterns can be assigned to this group. For the first type, one pair of line folds need to be co-axial. For the second type, the two symmetrical line folds have to be placed in parallel (figure 2*a*(ii)). Correspondingly, the internal rotating folds have relations either as (figure 2*c*, blue and red lines)


(3.17a) $$\theta_{x1}=\;\theta_{x2}\;or\;\theta_{y1}=\;\theta_{y2}\;or\;\theta_{z1}=\;\theta_{z2}$$

or (figure 2*c*, blue line)


(3.17b) $$\theta_{z1}=\;\theta_{z2}=\;\theta_{z3}=\;\theta_{z4}.$$

For the third group with two reconfiguration paths, each pair of line folds has to be co-axially and symmetrically placed, and the two pairs of line folds are perpendicular to each other (figure 2*a*(iii)(iv)). Thus, the fold rotation angles can be written as


(3.18) $$\theta_{x1}=\theta_{x2}\;and\;\;\theta_{y1}=\;\theta_{y2}.$$

From figure 2*b*, we can clearly see that structural units with more reconfiguration paths are those possessing fewer fold patterns. For example, group 3 has four combinatorial designs with two reconfiguration paths, while group 2 has 58 different cases with one reconfiguration path. We attribute this to the additional structural constraints induced from structural symmetries and the special spatial positions of the folds. Essentially, the specific fold arrangements of the structural unit with two reconfiguration paths provide the guideline of designing the hierarchical fold patterns when creating hierarchical building blocks, as illustrated in figure 2*d*.

Based on the 4-cube structural unit, we build the first building block for constructing the first type of hierarchical mechanical metamaterial. Figure 2*d,e* (left) illustrates the process of constructing the hierarchical building block, i.e. replacing the higher-level rigid links with the whole structure of the lower-level system. Note that the structural hierarchy strategy used here is not a strictly repeating pattern in each hierarchy level. We can mix and assign different types of looped kinematic mechanisms in different hierarchical structural levels. For instance, as demonstrated later, we construct the third-type hierarchical building block by using the 8R looped kinematic mechanism as a level-1 structure, while assigning the level-2 structure to the 4R looped kinematic mechanism.

The cube-shaped topology also allows combinatorial designs when constructing the hierarchical building blocks. However, rather than searching for all design possibilities, here we only consider those designs with structural symmetry constraints. Thus, based on the group 3 4-cube structural unit, we construct the 4-cube-based level-2 building blocks by applying similar structural symmetries when assigning the level-2 line folds. Through analysis, we find that the level-2 building block based on the second type of group 3 4-cube building block exhibits the most intriguing reconfiguration features, i.e.

1. Reconfiguring with kinematic bifurcations (figure 2*e*, middle and right);
2. Maintaining the looped structural topology during reconfiguration with expansion (figure 2*e*, configuration ①).

Figure 2*d* shows the construction details of the level-2 building block (with the purple cylindrical column representing the level-2 folds). Intrinsically, kinematic bifurcations occur either at the initial state or at configurations with the fold rotation angles equal to 45°. Referring to the singular value decomposition method, we can predict the location and occurrence of the kinematic bifurcations. First, we need to rewrite equation (3.14) into a more concise form:


(3.19) $$C_{n}\delta \theta_{En}=-E_{n},$$

where $C_{n}$ is the coefficient matrix, and *n* is a positive integer representing the *n*th prediction step. Singularly decomposing equation (3.19) gives


(3.20) $$C_{n}=S_{n}V_{n}D_{n}^{T},$$

where $S_{n}$ and $D_{n}$ are the two 4 × 4 and 4 × 4 square orthogonal matrices containing left- and right-singular vectors, respectively, while $V_{n}$ is a 4 × 4 rectangular matrix with non-zero singular values *v_ii_* on its main diagonal. Then, the occurrence and the location of kinematic bifurcations can be found by searching the zero singular value *v_ii_*.

Moreover, if assuming a unit length of the cube side, we have


(3.21) L=1,α=kπ/2,s=0,

where *k* = ‒4 to 4 is determined by the relative positions between the axes of two adjacent line folds (*z*-axis defined in figure 2*d* with positive direction right handed along the *x*-axes). Therefore, from equations (3.20) and (3.21), we can predict the kinematic bifurcation shown in figure 2*e* (*v*_11, 22, 33_ > 0 and *v*_44_ ≡ 0). Physically, this means that when the fold angles *θ*_*x*_ = *θ*_*y*_ = 45°, the building block can either reconfigure by following its original path from configuration ① to configuration ② (i.e. increasing *θ*_*x*_ and *θ*_*y*_ from 45° to 90°), or bifurcate to a path from configuration ① to configuration ③ shown in figure 2*e* (maintaining *θ*_*y*_ as 45° while increasing *θ*_*x*_ from 45° to 90°), middle and right.

Given the reconfigured structural topology of the level-2 building block, its simple expansion feature makes it ideal for constructing 3D mechanical metamaterials owing to the compatible deformation as demonstrated later.

### (c) 6-cube-based structural unit and the hierarchical building block #2

**Figure 3.**
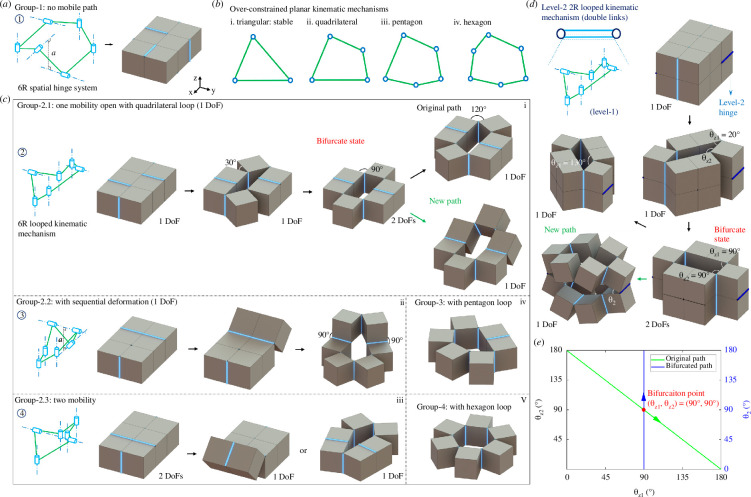
Construction of 6-cube-based structural unit and the second hierarchical building block. Beyond the non-reconfigurable designs (*a*) the construction of 6-cube-based building blocks have to be constructed as in-planar looped kinematic mechanisms (*b*) to reconfigure. Three groups of reconfigurable 6-cube-based building block are presented in (*c*). Based on the group 2-1, the hierarchical building block (*d*) is constructed with kinematic bifurcation (*e*).

Indicated by the trial-correction method, we note that it is impossible to reconfigure the 6-cube unit with out-of-plane shape morphing features owing to the conflict interactions among the rigid links (figure 3*a*, group 1). To overcome this issue, inspired by the planar looped kinematic mechanisms shown in figure 3*b*, such as the triangular, quadrilateral, pentagon and hexagon over-constrained planar kinematic mechanisms, we find that at least four folds are required to be arranged in parallel to create a planar reconfigurable kinematic mechanism. Thus, we create three different types of reconfigurable 6-cube-based building blocks based on the looped kinematic mechanisms in figure 3*b*.

For the first one with a quadrilateral loop, we present one optimal design with kinematic bifurcation. Figure 3*c–i* shows the details of the fold pattern and its equivalent 6R looped spatial kinematic mechanism (group 2-1). Given the in-plane reconfiguration features of the quadrilateral loop, we place the remaining twofolds on the same plane and in parallel to trigger the kinematic bifurcation. Thus, the group 2-1 6-cube structural unit can first reconfigure with a quadrilateral structural loop and then bifurcate when the fold rotation angle is 90°. At the bifurcated state, it either follows the original path to keep reconfiguring as a 4R planar looped kinematic mechanism or rotates out of plane along the new path as a 2R kinematic mechanism. In fact, this reconfiguration transition from in-plane state to out-of-plane state represents an efficient way of designing hierarchical mechanical metamaterials with programmable anisotropic structural stiffness. Enlightened by this, we create the level-2 building block shown in figure 3*d*.

In addition to group 2-1, we further construct two more reconfigurable groups using the 4R looped planar kinematic mechanism (groups 2-2 and 2-3, figure 3*c*). However, since no structural symmetries exist in their reconfigured profiles, they are less suitable to construct mechanical metamaterials when compared to group 2-1. Moreover, we also explore the design groups of the 6-cube building blocks reconfiguring as 5R (group−3) and 6R (group−4) looped kinematic mechanisms. However, owing to their solely in-plane reconfigurations and the incompatible boundaries for assembling periodic networks, these two design groups are also out of the scope of this work for creating hierarchical mechanical metamaterials.

As illustrated in figure 3*d*, the second hierarchical building block is constructed by assigning an over-constrained 2R looped kinematic mechanism on connecting level-2 folds. Particularly, the two level-2 folds are specially arranged with a relative spatial position to guarantee the inherence of both the reconfigurations and kinematic bifurcations of the level-1 6-cube unit. Remarkably, the level-2 structural unit exhibits similar reconfiguration features to its level-1 links, i.e. rotating as a quadrilateral loop first (figure 3*d*), then bifurcating when the level-1 fold rotation angles are 90° and lastly following two paths to change shapes with


(3.22) $$\begin{array}{c} \theta_{z1}+\;\theta_{z2}=90^{\circ }\;\left(original\;path\right)\\ \theta_{z1}=\;\theta_{z2}=90^{\circ },\;and\;\theta_{2}=0\;to\;180^{\circ }\;\left(new\;path\right), \end{array}\;$$

where $\theta_{z1}$ and $\theta_{z2}$ are the level-1 fold rotation angles, and $\theta_{2}$ is the level-2 fold rotation angle.

Based on this structural unit, we build another type of 3D bifurcated mechanical metamaterials as demonstrated later in the next section.

### (d) 8-cube-based structural unit and the hierarchical building block #3

**Figure 4.**
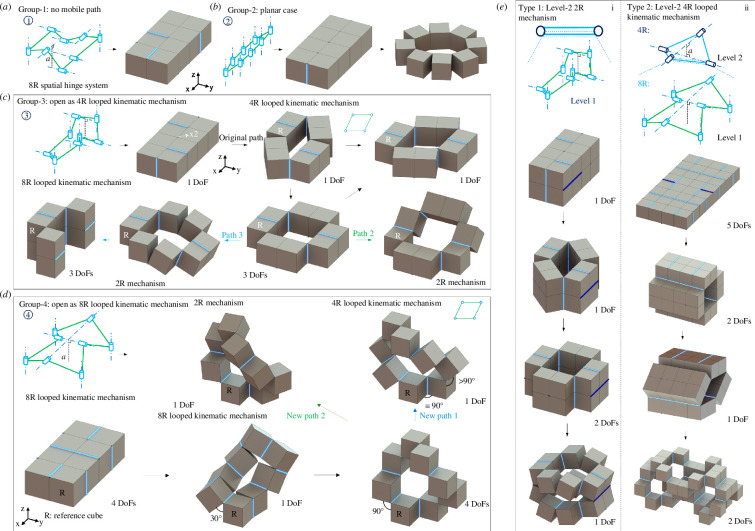
Construction of 8-cube-based structural unit and the third type of building block. Four groups of 8-cube structural units are constructed from (*a*) to (*d*). Two representative level-2 building blocks are constructed in (*e*).

Given the increasing number of cube structural elements, the construction of 8-cube structural units is more complicated. However, guided by the trial-correction method, we can still differentiate them into three groups (groups 1 to 3, figure 4) in terms of their reconfigurations features. The first group has specific fold arrangements without any reconfiguration paths. As shown in figure 4*a*, no structural symmetry exists for the line folds of group 1. The second group is constructed using 6-cube-based units to form the 8 line folds in the form of 4R, 5R, 6R, 7R and 8R in-plane looped kinematic mechanisms. Thus, this group of units can only reconfigure in plane (see the representative in figure 4*b* with all the eight folds rotated).

The third group exhibits similar reconfiguration features to the group 3 with 6-cube units. Figure 4*c* shows that fourfolds are arranged in parallel, enabling the structure reconfiguring first into a quadrilateral loop (4R looped planar mechanism). The remaining two pairs of folds are placed with twofold structural symmetry to trigger the occurrence of kinematic bifurcations when the other fourfolds rotate in plane with 90° (bottom-middle). Starting from the bifurcated point, two more reconfiguration paths occur (bottom-left and right) and the structure reconfigures as two chain mechanisms.

The fourth group is equipped with the largest number of structural symmetries and displays the most complicated reconfiguration features. Figure 4*d* shows that when spatially placing all the eightfolds into four symmetrical pairs, the fourth group 8-cube building block can bifurcate at its initial state. As demonstrated before [56], it reconfigures either as a 2R chain mechanism, or as a 6R spatial mechanism or as an 8R spatial mechanism. A new bifurcation point can occur when reconfiguring as an 8R spatial mechanism and evolve with two more reconfiguration paths (new path-1 and new path-2). The reconfiguration kinematics of this 8R structural unit has been thoroughly investigated in our previous work [55,56].

For the structural unit design, we refer to the hierarchical design strategy by using different kinematic mechanisms at different structural levels (figure 4*e*(i)). For simplicity, we build two hierarchical building blocks by using the simplest 2R and 4R looped kinematic mechanisms to connect level-2 folds. Based on the 8-cube units of groups 3 and 4, we create two divergent hierarchical building blocks, as shown in figure 4*e*. Since the first hierarchical building block reconfigures similarly to the 6-cube-based one, it is not discussed when describing the construction of bifurcated mechanical metamaterials.

By appropriately placing four level-2 folds (figure 4*e*(ii)), we build the third hierarchical building block, which can first reconfigure as a chain mechanism, and bifurcate when all the folds angles are 90°. After the bifurcation state, it can reconfigure to a new direction (path-2) and almost fully open all contacted structural elements (bottom). The construction of its related bifurcated mechanical metamaterials will be discussed in the next section.

## 4. Geometric mechanics of bifurcated hierarchical mechanical metamaterials

### (a) Construction of bifurcated hierarchical mechanical metamaterials

**Figure 5.**
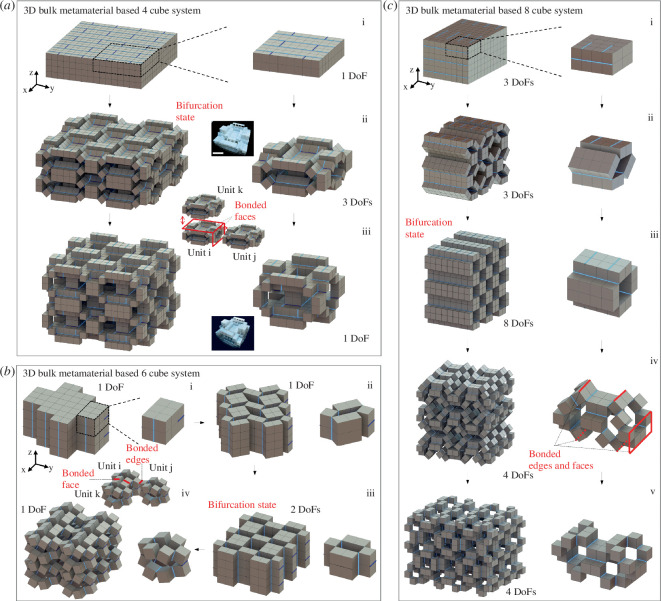
Three different types of bifurcated hierarchical mechanical metamaterials based on 4-cube building block (*a*), 6-cube building block (*b*) and 8-cube building block (*c*).

As illustrated in figure 5, we construct three different types of 3D bulk hierarchical mechanical metamaterials with bifurcated reconfiguration features. Note that the contact cube surfaces between the boundary cubes of building blocks are fully constrained to achieve uniform deformation. All three hierarchical mechanical metamaterials preserve the kinematic bifurcations of their building blocks and reconfigure into different configurations. In the following, we will study how the kinematic bifurcations affect the mechanical responses of these mechanical metamaterials.

### (b) Geometric mechanics

In this section, the geometric mechanics of the three bifurcated hierarchical mechanical metamaterials in figure 5 are investigated. For simplicity, we assume the torsional stiffness of level-1 and level-2 line folds as *K*_1_ and *K*_2_, respectively. During reconfiguration, the total elastic energy, *E,* stored in the hierarchical mechanical metamaterials can be expressed as


(4.1) $$E=E_{1}+E_{2}=E_{1}\left(\theta_{1}\right)+E_{2}\left(\theta_{2}\right),$$

where $E_{1}\left(\theta_{1}\right)$ and $E_{2}\left(\theta_{2}\right)$ are the elastic energy of level-1 and level-2 line folds, respectively.

Setting the deformed side length as


(4.2) $$u=u\left(\theta_{1},\theta_{2}\right),$$

we can derive the stretching stiffness as [35]


(4.3) $$k=\partial^{2}E/\partial u^{2}\left(\theta_{1},\theta_{2}\right).$$

Moreover, the Poisson’s ratio *ν_ij_* is defined as [35]


(4.4) $$v_{ij}=-\left(\frac{du_{i}}{u_{i}}\right)/\left(\frac{du_{j}}{u_{j}}\right),$$

with $u_{i}\;$ and $u_{j}\;$ being the displacement along *i* and *j* direction (*i*, *j* denotes the *x*, *y* or *z* direction), respectively.

For the first type of 4-cube-based hierarchical metamaterials, the level-1 and level-2 folds rotate with the same angle, i.e. $\theta_{1}=\theta_{2}$. Thus, equations (4.1) and (4.2) become


(4.5) $$E=N_{F}K{\left(\theta -\theta_{0}\right)}^{2}/2,$$

(4.6) $$\begin{array}{c} u_{x}=u_{y}=2a\left(cos\theta +sin\theta -1\right)\\ u_{z}=2a\left(sin\theta -cos\theta \right), \end{array}$$

with $N_{F1}$ being the total number of line folds and $\theta_{0}$ being the initial value of θ.

Combining equation (4.6) with equations (4.3) and (4.4), the structural stiffness *k_i_*, nominal strains *ε_ii_* and Poisson’s ratio *ν_ij_* can be derived as


(4.7) $$\begin{array}{c} k_{x}=k_{y}=N_{F1}\left[\left(cos\theta +sin\theta \right)\left(\theta -\theta_{0}\right)+cos\theta -sin\theta \right]/4a^{2}{\left(cos\theta -sin\theta \right)}^{3}\\ k_{z}=N_{F1}\left[\left(cos\theta -sin\theta \right)\left(\theta -\theta_{0}\right)-cos\theta -sin\theta \right]/4a^{2}{\left(cos\theta +sin\theta \right)}^{3}, \end{array}$$

(4.8) $$\begin{array}{c} \varepsilon_{xx}=\varepsilon_{yy}=\left(cos\theta +sin\theta -1\right)/4\\ \varepsilon_{zz}=sin\theta -cos\theta , \end{array}$$

(4.9) $$v_{xy}=-1,v_{xz}=v_{yz}={\left(cos\theta -sin\theta \right)}^{2}/4\left(cos\theta +sin\theta -1\right)\left(cos\theta +sin\theta \right),$$

where θ varies from 0° to 90°.

A similar derivation can be conducted for the second type of 6-cube-based and third type of 8-cube-based hierarchical mechanical metamaterials. For the 6-cube-based design, we have


(4.10) $$\begin{array}{c} k_{x1}=N_{F2}\left[\left(cos\theta_{z}+sin\theta_{z}\right)\left(\theta_{z}-\theta_{z0}\right)+cos\theta_{z}-sin\theta_{z}\right]/4a^{2}{\left(cos\theta_{z}-sin\theta_{z}\right)}^{3}\\ k_{y1}=N_{F2}\left[cos\theta_{z}+sin\theta_{z}\left(\theta_{z}-\theta_{z0}\right)\right]/4a^{2}{\left(cos\theta_{z}-sin\theta_{z}\right)}^{3},k_{z1}=k_{m}, \end{array}$$

(4.11) $$\begin{array}{c} \varepsilon_{xx}=2\left(cos\theta_{z}+sin\theta_{z}-1\right)/3\\ \varepsilon_{yy}=sin\theta_{z}\\ \varepsilon_{zz}=0, \end{array}$$

(4.12) $$v_{xy}=sin\theta_{z}\left(sin\theta_{z}-cos\theta_{z}\right)/cos\theta_{z}\left(sin\theta_{z}+cos\theta_{z}-1\right),v_{xz}=v_{yz}=0,$$

where $\theta_{z}$ increases from 0° to 90°; and


(4.13) $$\begin{array}{c} k_{y2}=\frac{N_{F2}\left[0.5sin0.5\theta_{x}-cos0.5\theta_{x}-\left(0.25cos0.5\theta_{z}+0.5sin0.5\theta_{z}\right)\left(\theta_{z}-\theta_{z0}\right)\right]}{4a^{2}{\left(0.5sin0.5\theta_{x}-cos0.5\theta_{x}\right)}^{3}}\\ k_{z2}=\frac{N_{F2}\left[\left(cos0.5\theta_{x}-0.5sin\theta_{x}\right)+\left(0.5cos0.5\theta_{x}+0.25sin0.5\theta_{x}\right)\left(\theta_{x}-\theta_{x0}\right)\right]}{4a^{2}{\left(cos0.5\theta_{x}-0.5sin0.5\theta_{x}\right)}^{3}},k_{x2}=k_{m}, \end{array}$$

(4.14) $$\begin{array}{c} \varepsilon_{xx}=0\\ \varepsilon_{yy}=0.5cos0.5\theta_{x}-sin0.5\theta_{x}\\ \varepsilon_{zz}=sin0.5\theta_{x}-0.5cos0.5\theta_{x}, \end{array}$$

(4.15) $$v_{yz}=\frac{\left(cos0.5\theta_{x}+2sin0.5\theta_{x}\right)\left(sin0.5\theta_{x}-0.5cos0.5\theta_{x}\right)}{\left(sin0.5\theta_{x}-0.5cos0.5\theta_{x}\right)\left(sin0.5\theta_{x}+2cos0.5\theta_{x}-1\right)},v_{xz}=v_{xy}=0,$$

where $\theta_{z}$ = 90° is fixed while $\theta_{x}$ increases from 0° to 180° and *k*_*m*_ is the material modulus of the folds.

**Figure 6.**
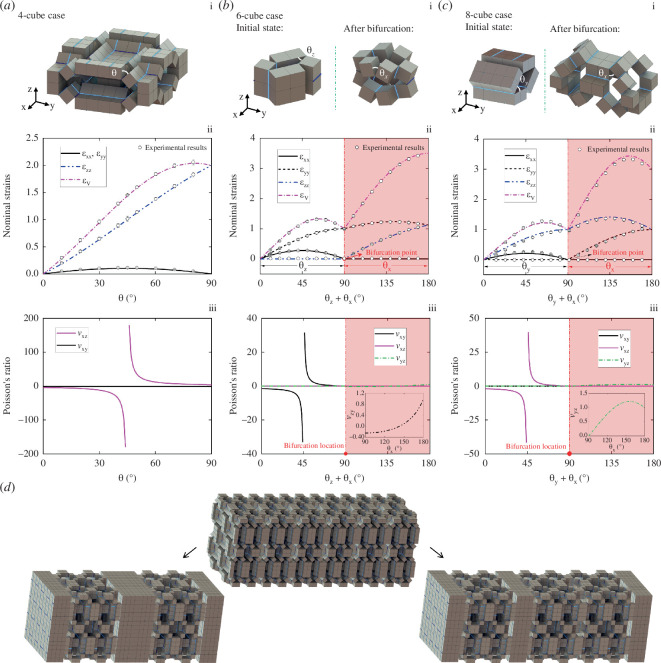
The mechanical properties and deformation features of the designed three different types of bifurcated hierarchical mechanical metamaterials. (*a*)–(*c*) the nominal strain (black dots: experimental results) and Poisson’s ratio. Remarkably, the first type can conduct with combinatorically reconfigured configuration states (*d*).

By comparison, we note that the second- and third-type hierarchical mechanical metamaterials exhibit similar reconfigurations and thus similar mechanical responses as verified by the results shown in figure 6*b,c*. For simplicity, here, we only show the final results of the deformation and mechanical properties of the second type of hierarchical mechanical metamaterials.

**Figure 7.**
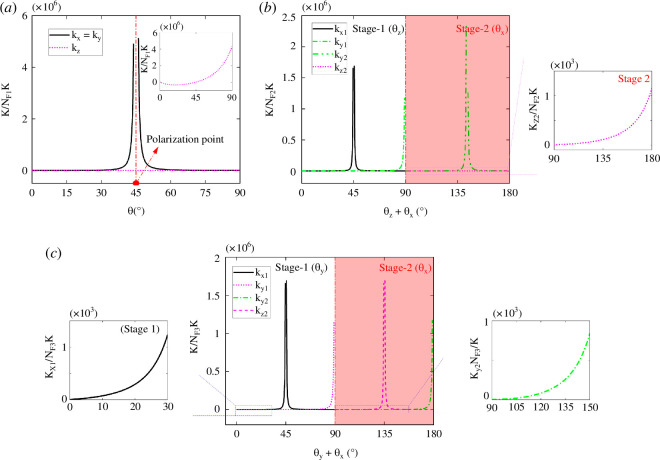
Structural stiffness analysis of the designed three different types of bifurcated hierarchical mechanical metamaterials based on 4-cube (*a*), 6-cube (*b*) and 8-cube structural units.


Figure 6*a*(ii) shows that the 4-cube-based hierarchical mechanical metamaterial expands first in the *xy*-plane when 0° ≤θ ≤ 45° ($\varepsilon_{xx}$ = $\varepsilon_{yy}$ > 0) and then shrinks when 45° ≤θ ≤ 90°. Meanwhile, the whole structure keeps expanding along the *z* direction ($\varepsilon_{zz}$ > 0). Overall, the structure always expands with $\varepsilon_{v}$ > 0. This expansion-to-shrink reconfiguration feature is induced by the intrinsic kinematic bifurcation of the hierarchical structural unit. It also acts as a singular/polarization point, in which the sign of the Poisson’s ratio $v_{xz}$ changes (figure 6*a*(iii)) from negative to positive, and the structural stiffnesses $k_{x}$, $k_{y}$ increase and then decrease (figure 7*a*). Moreover, given the symmetrical deformations of $\varepsilon_{xx}$ and $\varepsilon_{yy}$ from the kinematic bifurcation point, i.e.

(4.16) $$\varepsilon_{xx}\left(\theta \right)\equiv \varepsilon_{yy}\left(\theta \right)=\varepsilon_{xx}\left(90^{\circ }-\theta \right)\equiv \varepsilon_{yy}\left(90^{\circ }-\theta \right),$$

we can predict the bifurcated reconfiguration paths of the structural unit (figure 2*e*) in the first type of hierarchical mechanical metamaterials, which can combinatorically deform into divergent structural patterns. Based on a larger structure (figure 6*d*, middle), we demonstrate this by selectively bifurcating the structure into two deformed configurations: one is a configuration with the two ends and middle two segments reconfigured back to the initial compact state (figure 6*d*, left), and the other is a configuration with the two ends but only one internal segment closed (figure 6*d*, right). Note that given the single structural d.f. for each motion path and the limited number of reconfigured configurations, it is feasible to achieve all the desired shapes in the 6-cube- and 8-cube-based 3D metamaterials by uniaxial stretching. For the 4-cube-based case, its motion paths at the bifurcated configuration can exponentially increase with the number of structural units. However, we can still obtain the desired configurations through the boundary constraint method proposed in our recent work [63], where structural bistability can be endowed to the 3D structure by adding boundary structural constraints.

Comparing the results in figures 6*b,c* and 7*b,c*, the structural stiffness and the nominal strains of the second and third type of hierarchical mechanical metamaterials display almost identical variations. Before bifurcation, both two structures reconfigure in plane with one nominal strain increasing, while the other first increases and then decreases with the threshold located at $\theta_{z}$ = 45° (the second design) and $\theta_{x}$ = 45° (the third design). Reflected by the Poisson’s ratio, it decreases first to the threshold polarization point and then switches sign from negative to positive followed with continuous decrement (figure 6*b*,*c*(iii)). After passing bifurcation, the two hierarchical mechanical metamaterials can reconfigure from the *z*(*y*)-axis to the *x*(*x*)-axis, while the *x*-axis strain remains zero, and the other two strains increase to a maximum or increase first followed by a decrease. Interestingly, their Poisson’s ratio always stays as a relatively small positive value of no more than 1.5 (see insets, figure 6*b*, *c*(iii)).

Figure 7*b,c* shows that the structural stiffness exhibits obvious structural anisotropy influenced by intrinsic kinematic bifurcations. Remarkably, the structural stiffness of the second design along the *y*-axis exhibits two singular states, i.e. the first one at $\theta_{z}$ = 45° and $\theta_{x}$ = 0°, and the second one at $\theta_{z}$ = 90° and $\theta_{x}$ = 45°, as shown by the orange and brown lines in figure 7*b*. Similar phenomena can be observed in the first design (polarization switch of structural stiffness $k_{x}$ and $k_{y}$ at θ=45⁰). Thus, we can conclude that designing mechanical metamaterials with kinematic bifurcations can be a rather efficient strategy in programming structural anisotropy. To summarize, the advancement of our proposed bifurcated periodic structures includes two aspects: (i) triggered by kinematic bifurcation, the space of reconfigured shapes cam be exponentially enlarged by the rich combinations of the bifurcated paths and (ii) kinematic bifurcated metamaterials are capable of reconfiguring into multiple shapes through divergent bifurcated paths that exhibit ample and tunable features of structural stiffness and Poisson’s ratio.

## 5. Further remarks

In this article, based on the cube-based reconfigurable structural units, we construct a library of hierarchical building blocks and their assembly into 3D hierarchical mechanical metamaterials with kinematic bifurcations. A generic theoretical framework on predicting the reconfiguration kinematics and bifurcations is proposed. The structural hierarchy is a rather efficient method of creating structural units with rich reconfiguration features. The structural hierarchy can bring kinematic bifurcations into one over-constrained shape morphing mechanisms to increase the structural d.f., which in turn induces dramatic structural topology changes that can significantly tune the structural stiffness and Poisson’s ratio. We envision that our design strategy, of leveraging kinematic bifurcation to enrich the mechanical properties of mechanical metamaterials, can pave a new way in designing artificial structures/materials with novel properties and functionalities.

## Data Availability

All data needed to evaluate the conclusions are present in the manuscript and appendix.

## Appendix A: further discussion of kinematic bifurcation modelling

Here we discuss the kinematic bifurcation features of our proposed system with more than one d.f. by using a general but simple numerical method. Consider a closed-loop mechanism with n joints $\theta =\left[\theta_{1},\cdots ,\theta_{n}\right]$, its degree of freedom is m at the smooth part of the variety. In the joint space, if $\dim K_{1}>m$ at point $\theta_{p}$, this is an unsmooth point. The first- and second-order tangent cone ($K_{1}$ and $K_{2}$) at $\theta_{p}$ can be calculated using the screw theory. Other unsmooth points $\theta_{us}$ around $\theta_{p}$ can be found by solving equation $\sigma_{n-m+1}(\theta )=0$; $\sigma_{n-m+1}$ is the n-m+1 singular value and $K_{2}$ is used as an initial guess vector. Therefore, the bifurcation property around $\theta_{p}$ can be observed.

For example: implementing the above method in Group 4 in figure 4; the calculation result is shown below. The mechanism at the middle bifurcation state corresponds to an unsmooth point $\theta_{p}$ in joint space; $K_{2}$ is the union of two tangent planes. The unsmooth area $\theta_{us}$ is indeed a curve, which is a one-dimensional singularity. Therefore, this bifurcation occurs at the bifurcation line.

However, bifurcations may not only be one-dimensional, they are dependent on the complexity of the variety. For example, at point $\theta_{p}=\left[\pi ,\pi ,0,\pi ,\pi ,\pi ,0,\pi \right]$, the figure below shows that $K_{2}$ represents two coincident tangent planes. The neighbouring unsmooth points are located on a surface, which is a two-dimensional singularity.

Therefore, the topology around an unsmooth point is not a simple problem and not only depends on the dimension of the smooth manifolds, but also the geometry of the variety.
